# Effect of combination treatment with escitalopram and risperidone in punding behaviour among patients with dementia‐ a retrospective study

**DOI:** 10.1002/alz.088904

**Published:** 2025-01-09

**Authors:** Shailendra Mohan Tripathi, Mohit Kumar Shahi, Porimita Chutia

**Affiliations:** ^1^ University of Aberdeen, Aberdeen United Kingdom; ^2^ King George’s Medical University, Lucknow, Uttar Pradesh India

## Abstract

**Introduction:**

Punding is a unique stereotypical, repetitive, non‐goal‐directed behavior characterized by an intense fascination with daily objects. This behavior although commonly seen in dementia patients is relatively less studied. In this study, we aimed to assess the effectiveness of combination treatment of escitalopram and risperidone treatment in the punding behavior of dementia patients.

**Methodology:**

A retrospective study of dementia patients with punding behavior treated inpatient with a combination treatment of escitalopram and risperidone from January 2023 to October 2023. A semi‐structured proforma was used to collect the demographic details, illness information, and outcomes based on standardized rating scales from the records of a tertiary care center in India. The data were analyzed using paired t‐test and McNemar test.

**Results:**

A total of 29 patients, 17 females and 12 males with a mean age of 74.14 ± 7.18 years were included. A significant decline was found in the Neuropsychiatric Inventory (NPI) motor behavior subscale score and caregiver distress related to motor behavior from Day 0 to Day 7 (p‐value of 0.0002, p = 0.001) on the combination treatment. However, the total NPI Severity Score and total QUIP‐RP Score (for repetitive behavior) exhibited a reduction in scores from Day 0 to Day 7 but the p‐value was non‐significant.

**Conclusion:**

The study adds valuable insights into the management of punding behavior and its associated distress providing a basis for further research on the neurobiological underpinnings of this distinctive symptom in dementia.

